# Analysis of Neuropsychiatric Diagnoses After Montelukast Initiation

**DOI:** 10.1001/jamanetworkopen.2022.13643

**Published:** 2022-05-24

**Authors:** Tapio Paljarvi, Julian Forton, Sierra Luciano, Kimmo Herttua, Seena Fazel

**Affiliations:** 1 Department of Psychiatry, University of Oxford, Warneford Hospital, Headington, Oxford, England, United Kingdom; 2 Department of Forensic Psychiatry, University of Eastern Finland, Niuvanniemi Hospital, Kuopio, Finland; 3 Children’s Hospital for Wales, Heath Park, Cardiff, Wales, United Kingdom; 4 TriNetX, Cambridge, Massachusetts; 5 Department of Public Health, University of Southern Denmark, Esbjerg, Denmark; 6 Oxford Health National Health Service Foundation Trust, Warneford Hospital, Oxford, England, United Kingdom

## Abstract

**Importance:**

The evidence base for the association between montelukast and adverse neuropsychiatric outcomes is mixed and inconclusive. Several methodological limitations have been identified in the evidence base on the safety of montelukast in observational studies.

**Objective:**

To investigate the association between new montelukast exposure and 1-year incident neuropsychiatric diagnoses with improved precision and control for baseline confounders.

**Design, Setting, and Participants:**

This propensity score–matched cohort study was conducted using electronic health records from 2015 to 2019 in the TriNetX Analytics Network patient repository of more than 51 million patients from 56 health care organizations, mainly in the US. Included patients were those aged 15 to 64 years at index prescription for montelukast or for control prescription who had a history of asthma or allergic rhinitis. After propensity score matching for various baseline confounders, including comorbidities and dispensed prescription medicines, we included 154 946 patients, of whom 77 473 individuals were exposed to montelukast. Patients were followed up for 12 months. Data were analyzed from June through November 2021.

**Exposures:**

New dispensed prescription for leukotriene receptor antagonist montelukast or control medication.

**Main Outcomes and Measures:**

Incident neuropsychiatric diagnoses at 12 months identified using *International Statistical Classification of Diseases, Tenth Revision, Clinical Modification *(*ICD-10-CM*) codes.

**Results:**

There were 72 490 patients with asthma (44 726 [61.7%] women; mean [SD] age at index prescription, 35 [15] years) and 82 456 patients with allergic rhinitis (54 172 [65.7%] women; mean [SD] age at index prescription, 40 [14] years). In patients exposed to montelukast, the odds ratio [OR] for any incident neuropsychiatric outcome was 1.11 (95% CI, 1.04-1.19) in patients with asthma and 1.07 (95% CI, 1.01-1.14) in patients with allergic rhinitis compared with patients who were unexposed. The highest OR was for anxiety disorders (OR, 1.21; 95% CI, 1.05-1.20) among patients with asthma exposed to montelukast and insomnia (OR, 1.15; 95% CI, 1.05-1.27) among patients with allergic rhinitis exposed to montelukast.

**Conclusions and Relevance:**

This study found that patients with asthma or allergic rhinitis had increased odds of adverse neuropsychiatric outcomes after montelukast initiation. These findings suggest that clinicians should consider monitoring potential adverse mental health symptoms during montelukast treatment, particularly in individuals with a history of mental health or sleep problems.

## Key Points

**Question:**

Is montelukast associated with adverse neuropsychiatric outcomes?

**Findings:**

In this cohort study of electronic health records for 72 490 patients with asthma and 82 456 patients with allergic rhinitis, montelukast was associated with higher odds of incident neuropsychiatric outcomes, including anxiety and insomnia.

**Meaning:**

This finding suggests that clinicians should consider monitoring for potential mental health symptoms during montelukast treatment.

## Teaser

This cohort study investigates the association of montelukast treatment with adverse neuropsychiatric outcomes among patients with asthma or allergic rhinitis.

## Introduction

Montelukast, the most widely used leukotriene-modifying agent (LTMA), is a selective leukotriene receptor antagonist and is currently indicated for prophylactic and chronic treatment of asthma, relief of symptoms of allergic rhinitis, and acute prevention of exercise-induced bronchoconstriction. Off-label uses include for chronic obstructive pulmonary disease and obstructive sleep apnea.^1,2^ There is also increasing interest in repositioning montelukast for treating various other health conditions.^3,4,5,6^

The evidence base for adverse neuropsychiatric outcomes associated with LTMAs is mixed and inconclusive.^7^ Nevertheless, in 2020, the US Food and Drug Administration (FDA) issued a new warning about potential serious mental health outcomes associated with montelukast to promote further awareness of potential adverse effects and also advised reducing montelukast’s use in the treatment of allergic rhinitis.^8^ Drug safety updates on montelukast have been published recently in Europe.^9^ On one hand, controlled clinical trials have reported mainly mild and infrequent adverse effects.^10^ This led to an initial conclusion that montelukast was a safe and well-tolerated medicine.^11,12,13,14,15^ Contrasting evidence, however, has come from postmarketing safety signals, which have included a range of severe neuropsychiatric outcomes, such as aggression, anxiety, depression, various sleep-related problems, and suicidal ideation, self-harm, and completed suicide.^7,16,17,18,19,20,21,22^ Some case studies have reported a positive montelukast dechallenge-rechallenge association, that is, that adverse symptoms have resolved after cessation of treatment and returned after restarting of treatment.^7,23^ Evidence from observational studies, which are often methodologically better suited for studying adverse outcomes, is also mixed and inconclusive.^7^ Several methodological limitations have been identified in the evidence base on the safety of montelukast in observational studies, including potential confounding by indication, comorbidities of asthma, and concurrent use of other medicines.^7,24^ Furthermore, because much of the evidence is based on pediatric and adolescent populations, relatively little is known about potential adverse outcomes among adults.

To address some of the shortcomings of the current evidence base on potential adverse outcomes associated with montelukast, we conducted a propensity score–matched 1-year follow-up study of incident neuropsychiatric diagnoses among patients with a new treatment episode with montelukast. We used a patient repository of more than 51 million patients. Of LTMAs currently available, we focused on montelukast because 99% of patients with prescriptions for LTMAs in our data were prescribed montelukast. Furthermore, because of the FDA’s recent recommendation on reducing use of montelukast in treating allergic rhinitis, we examined incident neuropsychiatric outcomes separately for patients with asthma and allergic rhinitis.

## Methods

Because this cohort study used deidentified patient records and did not involve the collection, use, or transmittal of individually identifiable data, this study was exempt from institutional review board approval and informed consent based on the Health Insurance Portability and Accountability Act (eMethods in the Supplement). This study follows the Strengthening the Reporting of Observational Studies in Epidemiology (STROBE) reporting guideline.

We used the TriNetX proprietary patient repository and analytics platform (TriNetX) to identify eligible patients between the years 2015 and 2019. The TriNetX Analytics Network platform provided access to pseudonymized, aggregate-level information from electronic health care records (EHRs), including information on diagnoses and dispensed medicines, of more than 51 million patients aged 15 to 64 years from 56 health care organizations, mainly in the US (with 91% of patients in the US and 9% in Europe, Latin America, or the Asia-Pacific region). See eMethods in the Supplement for further details on data and methods.

Using the TriNetX Analytics Network, we identified a relatively large number of patients who had at least 1 dispensed prescription for montelukast (450 078 individuals). Patients were eligible if they were aged 15 to 64 years at index prescription; had diagnosed asthma or allergic rhinitis; did not have a diagnosis for other chronic obstructive pulmonary disease, obstructive sleep apnea, or neoplasms; and were alive for the subsequent 12 months after the index prescription. We excluded patients who were identified to be pregnant during the study period. Patients were followed up for 12 months after their index prescription for the selected neuropsychiatric diagnoses.

### Cohort Definitions

We defined 2 separate study populations: patients with asthma and patients with allergic rhinitis. From the allergic rhinitis groups, we excluded patients who had a history of diagnosed asthma or who received an asthma diagnosis during the follow-up period to study the association independent of diagnosed asthma. Because allergic rhinitis is a common comorbidity of asthma, we did not exclude patients with diagnosed allergic rhinitis from asthma groups.

Patients in control groups did not have dispensed prescriptions for montelukast 6 months before the index prescription or during follow-up. We identified 475 869 patients fulfilling eligibility criteria before matching, of whom 276 413 individuals were included in the asthma population and 199 456 individuals were included in the allergic rhinitis population (eFigures 1 and 2 in the Supplement, respectively).

#### Asthma Groups

To include only individuals who received treatment for asthma, we included patients with asthma who had an asthma-related health care contact during a given study entry year. Each study year, we used the first dispensed montelukast prescription in the data as the index prescription for patients with asthma exposed to montelukast, whereas for the asthma control group, we used the first dispensed prescription for inhaled corticosteroids or inhaled bronchodilators, whichever came first, as the index prescription. To partly control for confounding by unstable or poorly controlled asthma, we excluded patients with asthma who had dispensed prescriptions for oral glucocorticoids during the study entry year or during follow-up.

#### Allergic Rhinitis Groups

To include only individuals who received treatment for allergic rhinitis, we included patients who had a health care contact related to allergic rhinitis (diagnoses for allergic rhinitis due to pollen, other seasonal allergic rhinitis, other allergic rhinitis, or unspecified allergic rhinitis) during a given study year. Each study year, we used the first dispensed montelukast prescription in the data as the index prescription for patients exposed to montelukast, whereas for the control group, we used the first dispensed prescription for any of 3 most prescribed nonsedative antihistamines in the data (cetirizine, fexofenadine, or loratadine) as the index prescription. To partly control for confounding by decreased lung function, we excluded patients with allergic rhinitis who had diagnosed dyspnea.

### Propensity Score Matching

For propensity score matching, we used the TriNetX built-in algorithm, which was based on 1:1 nearest-neighbor matching with a caliper of 0.1 SD. For matching, we included various covariates on demographic characteristics, history of neuropsychiatric diagnoses, physical comorbidities, and dispensed prescription drugs, which were recorded any time before the index prescription. Please see eMethods in the Supplement for the full list of covariates. The same set of covariates were used to match montelukast-exposed and -unexposed groups among patients with asthma and allergic rhinitis; however, for patients with asthma, type of asthma diagnosis (ie, mild intermittent asthma, mild persistent asthma, moderate persistent asthma, severe persistent asthma, or other or unspecified asthma) was also included. Type of asthma was not included as a covariate among patients with allergic rhinitis because patients with asthma were excluded from this population. The medical codes used to identify these conditions and dispensed prescription medicines are shown in eTable 1 in the Supplement. After propensity score matching, we included 154 946 patients (72 490 patients diagnosed with asthma and 82 456 patients diagnosed with allergic rhinitis), of whom 77 473 individuals were exposed to montelukast. Cohorts were balanced for all included covariates; that is, all standard differences were less than 0.1 after propensity score matching (eTables 2 and 3 in the Supplement).

### Outcome Measurement

Primary outcome measures were 12-month incident neuropsychiatric diagnoses identified by *International Statistical Classification of Diseases, Tenth Revision, Clinical Modification *(*ICD-10-CM*) codes, including psychotic disorders; mood disorders; anxiety, dissociative, stress-related, somatoform, and other nonpsychotic mental disorders; adult personality and behavior disorders; sleep disorders; and nonfatal self-harm, which also included events of undetermined intent. Within these broader diagnostic groups, we also looked at more specific incident diagnoses, including manic episode or bipolar disorder; major depression, single episode; phobic anxiety; generalized anxiety; other anxiety disorders; obsessive compulsive disorder and behavior (OCD); insomnia and sleep deprivation; hypersomnia; circadian rhythm disorders; parasomnias, including sleepwalking, sleep terrors, and nightmare disorder; sleep-related movement disorders and restless legs syndrome; and other or unspecified sleep disorders.

Secondary outcomes were used to triangulate primary outcomes; that is, they were used as proxies for diagnoses of clinically relevant neuropsychiatric outcomes receiving treatment. These secondary outcomes were 12-month incident dispensed prescriptions for psychotropic medicines, including sedatives and hypnotics, antidepressants, antipsychotics, and medicines commonly used in the US to treat sleep problems, such as insomnia (doxepin, estazolam, eszopiclone, flurazepam, melatonin, suvorexant, temazepam, trazodone, triazolam, ramelteon, zaleplon, and zolpidem). The RxNorm codes used to identify these outcomes are shown in eTable 1 in the Supplement. Furthermore, 12-month emergency visits and inpatient visits were included as proxy outcomes for general health status.

### Statistical Analysis

For 12-month incident outcomes, we calculated incidence rates per 1000 individuals and odds ratios (ORs) with 95% CIs for each of 5 baseline years separately (2015-2019). We used the Mantel-Haenszel method with random effects to obtain a weighted pooled OR over the 5-year study period. ReviewManager software version 5.4 (Cochrane) was used to estimate pooled ORs. Statistical significance was defined as 95% CIs not crossing 1. Data were analyzed June through November 2021.

## Results

There were 72 490 patients with asthma (44 726 [61.7%] women; mean [SD] age at index prescription, 35 [15] years) and 82 456 patients with allergic rhinitis (54 172 [65.7%] women; mean [SD] age at index prescription, 40 [14] years). Patients in the montelukast-exposed asthma group had higher odds (OR, 1.13; 95% CI, 1.02-1.25) of being diagnosed with any incident sleep disorder within 1 year of initial montelukast prescription compared with patients with asthma who were unexposed (Table 1). An association was also seen for patients with allergic rhinitis. Patients in the montelukast-exposed allergic rhinitis group had higher odds (OR, 1.10; 95% CI, 1.01-1.20) of being diagnosed with any incident sleep disorder within a year of initial montelukast prescription compared with patients in the unexposed allergic rhinitis group. In asthma and allergic rhinitis groups, montelukast exposure was associated with 13% to 15% higher odds of receiving an incident diagnosis for insomnia within 12 months after the initial montelukast prescription compared with no exposure (asthma: OR, 1.13; 95% CI, 1.01-1.27; allergic rhinitis: OR, 1.15; 95% CI, 1.05-1.27) (Table 1).

**Table 1.** One-Year Incidence of Sleep Outcomes

Sleep outcome	Exposed			Unexposed			OR (95% CI)
	Patients in group, No.	Patients with outcome, No.	IR, No./1000 individuals^a^	Patients in group, No.	Patients with outcome, No.	IR, No./1000 individuals^a^	
**Patients with asthma**							
Total, No.	36 245	NA	NA	36 245	NA	NA	NA
Any sleep problem	32 677	817	25	32 814	729	22	1.13 (1.02-1.25)
Insomnia	33 512	628	19	33 725	558	16	1.13 (1.01-1.27)
Hypersomnia	35 970	85	2	35 998	80	2	1.06 (0.78-1.44)
Circadian rhythm disorder	36 140	50	1	36 108	50	1	1.00 (0.67-1.48)
Parasomnia	36 152	52	1	36 109	50	1	1.04 (0.70-1.53)
Movement disorder	35 919	85	2	35 901	80	2	1.06 (0.78-1.44)
Other and undefined sleep disorder	35 425	184	5	35 340	167	5	1.10 (0.89-1.36)
**Patients with allergic rhinitis**							
Total, No.	41 228	NA	NA	41 228	NA	NA	NA
Any sleep problem	36 847	1131	31	36 987	1033	28	1.10 (1.01-1.20)
Insomnia	37 505	926	25	37 710	810	21	1.15 (1.05-1.27)
Hypersomnia	40 984	93	2	40 970	90	2	1.04 (0.77-1.39)
Circadian rhythm disorder	41 064	50	1	41 077	53	1	0.94 (0.64-1.39)
Parasomnia	41 105	51	1	41 091	41	1	1.15 (0.69-1.90)
Movement disorder	40 769	118	3	40 785	101	2	1.16 (0.85-1.58)
Other and undefined sleep disorder	40 646	231	6	40 544	257	6	0.90 (0.75-1.07)

Abbreviations: IR, incidence rate; NA, not applicable; OR, odds ratio.

^a^ Incidence rate is incidents per 1000 individuals among patients aged 15 to 64 years at index prescription in years 2015 to 2019 after matching. The same patient could contribute to more than 1 incident diagnosis from different diagnostic groups during follow-up.

Patients in the montelukast-exposed asthma group had higher odds (OR, 1.21; 95% CI, 1.05-1.20) of being diagnosed with any incident anxiety-related disorder within a year of initial montelukast prescription compared with patients who were unexposed (Table 2). Associations for mental health outcomes were also seen for patients with allergic rhinitis. Patients in the montelukast-exposed allergic rhinitis group had higher odds (OR, 1.12; 95% CI, 1.05-1.19) of being diagnosed with any incident anxiety-related disorder within a year of initial montelukast prescription compared with patients who were unexposed. Incident major depression (single episode) was frequently diagnosed within 12 months of initial montelukast exposure (eg, 32 diagnoses per 1000 individuals among patients with asthma who were exposed), but no statistically significant difference was observed between the montelukast-exposed and -unexposed groups. For nonfatal self-harm, the difference in incidence rate between patients who were and were not exposed to montelukast was 3 diagnoses per 1000 individuals, but the OR was statistically nonsignificant (1.06; 95% CI, 0.72-1.55). There was no difference in incidence rate for nonfatal self-harm between patients with allergic rhinitis who were exposed vs not exposed. In patients with asthma and allergic rhinitis, montelukast exposure was associated with approximately 16% to 17% higher odds of receiving an incident prescription for antidepressants during follow-up compared with patients who were unexposed (asthma: 1.16; 95% CI, 1.07-1.26; allergic rhinitis: OR, 1.17; 95% CI, 1.05-1.30) (Table 3). Overall, montelukast exposure was associated with an excess of incident neuropsychiatric diagnoses compared with no exposure in patients with asthma (OR, 1.11; 95% CI, 1.04-1.19) and those with allergic rhinitis (OR, 1.07; 95% CI, 1.01-1.14) (Table 4).

**Table 2.** One-Year Incidence of Mental Health Outcomes

Mental health outcome	Exposed			Unexposed			OR (95% CI)
	Patients in group, No.	Patients with outcome, No.	IR, No./1000 individuals^a^	Patients in group, No.	Patients with outcome, No.	IR, No./1000 individuals^a^	
**Patients with asthma**							
Total, No.	36 245	NA	NA	36 245	NA	NA	NA
Psychotic disorder	35 767	79	2	35 739	74	2	1.07 (0.78-1.47)
Mood disorder	28 842	1104	38	28 971	1042	36	1.07 (0.98-1.16)
Manic episode or bipolar disorder	35 134	157	4	35 094	150	4	1.05 (0.84-1.31)
Major depression, single episode	30 495	986	32	30 673	947	31	1.05 (0.96-1.15)
Anxiety or related disorder	27 056	1732	64	27 088	1555	57	1.21 (1.05-1.20)
Phobic anxiety	35 873	115	3	35 882	102	3	1.13 (0.86-1.48)
Generalized anxiety	34 095	618	18	33 992	523	15	1.18 (1.05-1.33)
Other anxiety	29 233	1396	48	29 213	1261	43	1.11 (1.02-1.21)
Obsessive-compulsive disorder and behavior	35 975	65	2	35 955	56	1	1.15 (0.79-1.68)
Adult personality disorder	35 536	102	3	35 594	100	3	1.02 (0.77-1.35)
Self-harm, nonfatal	36 047	55	1	36 048	52	1	1.06 (0.72-1.55)
**Patients with allergic rhinitis**							
Total, No.	41 228	NA	NA	41 228	NA	NA	NA
Psychotic disorder	40 930	52	1	40 912	68	2	0.77 (0.53-1.10)
Mood disorder	34 251	1310	38	34 076	1286	38	1.02 (0.90-1.17)
Manic episode or bipolar disorder	40 527	120	3	40 535	129	3	0.94 (0.66-1.34)
Major depression, single episode	35 820	1120	31	35 603	1116	31	1.00 (0.91-1.10)
Anxiety or related disorder	31 507	2205	70	31 610	1996	63	1.12 (1.05-1.19)
Phobic anxiety	40 865	105	2	40 831	122	3	0.86 (0.66-1.12)
Generalized anxiety	38 731	785	20	38 833	692	18	1.14 (1.00-1.30)
Other anxiety	33 855	1730	51	33 724	1595	47	1.09 (0.98-1.22)
Obsessive-compulsive disorder and behavior	40 927	57	1	40 915	54	1	1.06 (0.73-1.35)
Adult personality disorder	40 816	69	2	40 781	80	2	0.86 (0.62-1.20)
Self-harm, nonfatal	41 067	51	1	41 043	51	1	1.00 (0.68-1.47)

Abbreviations: IR, incidence rate; NA, not applicable; OR, odds ratio.

^a^ Incidence rate is incidents per 1000 individuals among patients aged 15 to 64 years at index prescription in years 2015 to 2019 after matching. The same patient could contribute to more than 1 incident diagnosis from different diagnostic groups during follow-up.

**Table 3.** One-Year Incidence of Psychotropic Prescription Medicines for Sleep or Mental Health Problems

Drug	Exposed			Unexposed			OR (95% CI)
	Patients in group, No.	Patients with outcome, No.	IR, No./1000 individuals^a^	Patients in group, No.	Patients with outcome, No.	IR, No./1000 individuals^a^	
**Patients with asthma**							
Total, No.	36 245	NA	NA	36 245	NA	NA	NA
Psychotropic drug^b^	21 460	1826	85	22 126	1698	77	1.12 (1.04-1.20)
Sedative or hypnotic	27 629	1221	44	27 779	1177	42	1.05 (0.96-1.13)
Antidepressant	25 522	1519	59	26 489	1372	52	1.16 (1.07-1.26)
Antipsychotic	34 117	311	9	34 218	345	10	0.91 (0.75-1.11)
Sleep medication^c^	32 139	629	19	32 324	569	18	1.11 (0.99-1.25)
**Patients with allergic rhinitis**							
Total, No.	41 228	NA	NA	41 228	NA	NA	NA
Psychotropic drug^b^	25 274	3151	125	25 619	2969	116	1.08 (0.99-1.19)
Sedative or hypnotic	31 772	2417	76	32 001	2366	74	1.03 (0.97-1.09)
Antidepressant	29 779	2329	78	30 286	2043	67	1.17 (1.05-1.30)
Antipsychotic	39 906	390	10	39 770	409	10	0.95 (0.81-1.12)
Sleep medication^c^	36 906	887	24	36 962	856	23	1.04 (0.94-1.14)

Abbreviations: IR, incidence rate; NA, not applicable; OR, odds ratio.

^a^ Incidence rate is incidents per 1000 individuals among patients aged 15 to 64 years at index prescription in years 2015 to 2019 after matching. The same patient could contribute to more than 1 incident diagnosis from different diagnostic groups during follow-up.

^b^ Any of the selected groups of medicines, including sedatives and hypnotics, antidepressants, antipsychotics, and sleep medications.

^c^ Sleep medications include doxepin, estazolam, eszopiclone, flurazepam, melatonin, suvorexant, temazepam, trazodone, triazolam, ramelteon, zaleplon, and zolpidem.

**Table 4.** Additional Outcomes

Outcome	Exposed			Unexposed			OR (95% CI)
	Patients in group, No.	Patients with outcome, No.	IR, No./1000 individuals^a^	Patients in group, No.	Patients with outcome, No.	IR, No./1000 individuals^a^	
**Patients with asthma**							
Total, No.	36 245	NA	NA	36 245	NA	NA	NA
Any neuropsychiatric outcome^b^	22 877	1850	81	22 998	1683	73	1.11 (1.04-1.19)
Visits^c^							
Emergency	36 245	3714	NA	36 245	4167	NA	0.88 (0.83-0.93)
Inpatient	36 245	2068	NA	36 245	1988	NA	1.05 (0.95-1.15)
**Patients with allergic rhinitis**							
Total, No.	41 228	NA	NA	41 228	NA	NA	NA
Any neuropsychiatric outcome^b^	26 962	2398	89	27 026	2259	84	1.07 (1.01-1.14)
Visits^c^							
Emergency	41 228	3801	NA	41 228	4485	NA	0.85 (0.83-0.93)
Inpatient	41 228	2617	NA	41 228	3081	NA	0.95 (0.85-1.15)

Abbreviations: IR, incidence rate; NA, not applicable; OR, odds ratio.

^a^ Incidence rate is incidents per 1000 individuals among patients aged 15 to 64 years at index prescription in years 2015 to 2019 after matching. The same patient could contribute to more than 1 incident diagnosis from different diagnostic groups during follow-up.

^b^ One-year incident outcomes.

^c^ Including patients with history of emergency or inpatient visits.

Models for primary outcomes before matching showed different changes in odds and even opposite associations compared with models after matching (eTables 4 and 5 in the Supplement). For example, ORs for any incident anxiety-related disorder among patients who were exposed were 0.98 (95% CI, 0.93-1.02) for patients with asthma and 1.07 (95% CI, 1.00-1.15) for patients with allergic rhinitis compared with those who were not exposed. This suggests substantial confounding by included covariates, particularly for mental health outcomes. Before matching, patients exposed to montelukast had statistically significantly lower odds for incident psychotic disorders, mood disorders, and adult personality disorders compared with patients who were not exposed.

## Discussion

In this cohort study of 77 473 adult patients exposed to montelukast, we investigated the association between a new treatment episode with montelukast and incident neuropsychiatric diagnoses over the subsequent year, separately in individuals with diagnosed asthma and those diagnosed with allergic rhinitis. After propensity score matching, patients exposed to montelukast had a statistically significant excess of incident generalized anxiety disorder, insomnia, and prescriptions for antidepressants compared with patients not exposed to montelukast. Associations were seen in patients diagnosed with asthma and patients with allergic rhinitis who did not have diagnosed asthma. Overall, there were higher odds of experiencing any incident neuropsychiatric outcome within a year from the initial montelukast prescription compared with patients not exposed to montelukast. After controlling for various potential baseline confounders by propensity score matching, no statistically significant associations were observed for other major neuropsychiatric outcomes, such as psychotic disorders, mood disorders, OCD, or nonfatal self-harm. These findings suggest that future research should aim to identify potential predisposing factors or triggers associated with neuropsychiatric outcomes during montelukast treatment.

Although a meta-analysis^10^ of 46 randomized controlled trials did not find an association between montelukast exposure and neuropsychiatric outcomes (OR, 1.14; 95% CI, 0.94-1.39), its subgroup analyses found that patients with asthma who were exposed to montelukast had an excess of neuropsychiatric outcomes, as did patients with seasonal allergic rhinitis exposed to montelukast, but this was not statistically significant. No OR for sleep disturbances was reported, but as calculated using data provided by the authors, this may have been statistically significant (OR, 1.39; 95% CI, 1.02-1.90). Our findings are in line with these results concerning the relative contribution of various neuropsychiatric outcomes, but in contrast, our ORs were statistically significant in patients with asthma and those with allergic rhinitis. While randomized controlled trials are less prone to various sources of bias compared with observational studies, including confounding by indication, our propensity score–matched observational study benefitted from a large number of patients, with associated precision of estimates for these rare outcomes. We also found an association between montelukast exposure and incident prescriptions for antidepressants among patients with asthma and those with allergic rhinitis. Because we did not have information on diagnoses for which antidepressants were prescribed, we were not able to establish if antidepressants were prescribed for primary insomnia, anxiety, or depression. Associations between montelukast and antidepressant prescriptions have been reported in other observational studies.^25,26^

However, the interpretation of the observed population-level association between montelukast exposure and anxiety and depression–related symptoms is complicated by several factors. First, we cannot make conclusions on causality in an observational study; additionally, there is no potential biological mechanism for montelukast-associated neuropsychiatric outcomes.^27^ Second, there is a lack of other high-quality observational studies with large enough sample sizes, appropriate control for confounding, or detailed measurement of potential adverse outcomes. In a 2018 narrative review of 33 studies,^7^ 2 of 6 observational studies found an association between montelukast and neuropsychiatric outcomes. A 2021 observational study^24^ analyzed US data of more than 457 000 patients with asthma and found that, after propensity score matching, montelukast exposure was associated with a decreased risk of outpatient depression. We similarly observed an association with incident major depression among patients with asthma who were exposed to montelukast, but the association was observed only before propensity score matching and not after matching for various potential baseline confounders. We observed similar indications suggesting confounding for various other neuropsychiatric outcomes when comparing results before and after matching.

### Strengths and Limitations

A major strength of our data was the relatively large number of patients initially prescribed montelukast. This allowed us to investigate associations between montelukast and various neuropsychiatric outcomes with improved precision and control for baseline confounders, establish associations separately for patients with asthma and those with allergic rhinitis, and examine associations for various different neuropsychiatric outcomes (ie, to triangulate outcomes).

This study also has several limitations. Because we used retrospective EHR data, we did not have control over treatment allocation. Our results thus represent treatment decisions made in the clinic. We did not have information on the duration of montelukast treatment or adherence, which may have confounded observed associations. Among patients in a clinical setting, there may be considerable delay in seeking care for neuropsychiatric symptoms, particularly if symptoms are mild initially, which may obscure the correct sequence of events. We aimed to partly control for this by using a 14-day washout period after index prescription for measuring incident outcomes to reduce potential bias from conditions that were already present at the time of index prescription. This may have underestimated associations. Inclusion of patients with past montelukast exposures (ie, potential montelukast exposure earlier than the prior 6 months before index prescription) may have also underestimated observed associations because past use is likely associated with better tolerability of montelukast and thus decreased likelihood of adverse effects during follow-up. We focused on new treatment episodes with montelukast to reduce bias from exposure to LTMAs before the index prescription. Outcomes may be different among patients with continuous long-term exposure to montelukast. Because we used EHR data to identify neuropsychiatric outcomes, we identified only patients receiving treatment for these conditions. This has likely further underestimated the number of patients experiencing these outcomes because patients with less severe symptoms may not have been identified. This suggests that future observational research should investigate incidence of milder symptoms, in particular for sleep problems. By matching for various comorbidities and other prescription medicines separately by montelukast indication, we aimed to control for confounding by indication and potential differences in baseline health status. However, it is possible that some residual confounding by indication remained in the models for unmeasured risk factors not captured by EHR data, such as confounding by differences in disease progression and symptom severity.^28^ EHR data do not include information on all potential confounders, such as unemployment or living alone, which is a limitation in all studies using EHR data. If patients received treatment from health care organizations not participating in TriNetX before or after index prescription, this may have introduced bias owing to misclassification in exposure status or loss to follow-up for outcomes. We did not have information on mortality, except for inpatient deaths. This suggests that further research with similarly improved design is needed to investigate the association between montelukast and cause-specific mortality.

## Conclusions

We observed a population-level association between new montelukast exposure and incident neuropsychiatric diagnoses within a subsequent year of the initial dispensed prescription among patients with asthma or allergic rhinitis compared with propensity score-matched patients not exposed to LTMAs. The association was mainly explained by an excess incidence of anxiety and insomnia in patients exposed to montelukast. The statistically significant excess of incident neuropsychiatric outcomes in patients prescribed montelukast was small in absolute terms. However, because montelukast is prescribed to several million patients in the US each year, a small excess risk can be relevant at a population level. Future research with representative samples should quantify the total population burden of potential montelukast-associated neuropsychiatric outcomes. Our findings suggest that clinicians prescribing montelukast and other LTMAs should discuss potential neuropsychiatric adverse effects with patients before starting treatment and consider monitoring for signs of neuropsychiatric symptoms during treatment regardless of treatment indication. This may be particularly important in individuals who have a history of mental health or sleep problems.

## Notes

Supplement.
eMethods.

eReferences.
**eTable 1.** Medical Codes Used in Defining Cohorts, Propensity Score Matching, and Defining Outcomes **eFigure 1.** Flow Diagram of Montelukast-Exposed and -Unexposed Asthma Groups **eFigure 2.** Flow Diagram of Montelukast-Exposed and -Unexposed Allergic Rhinitis Groups **eTable 2.** Baseline Characteristics of Patients in Asthma Groups Before and After Matching by Exposure to Montelukast **eTable 3.** Baseline Characteristics of Patients in Allergic Rhinitis Groups Before and After Matching by Exposure to Montelukast **eTable 4.** 1-Year Incidence of Sleep Outcomes Before Matching **eTable 5.** 1-Year Incidence of Mental Health Outcomes Before Matching
