# Supplementary material for: Analysis of Neuropsychiatric Diagnoses After Montelukast Initiation
Source: JAMA Netw Open. 2022 May 24;5(5):e2213643. doi: 10.1001/jamanetworkopen.2022.13643 (PMC9131741; doi:10.1001/jamanetworkopen.2022.13643)
Supplement: Supplement. — eMethods. eReferences. eTable 1. Medical Codes Used in Defining Cohorts, Propensity Score Matching, and Defining Outcomes eFigure 1. Flow Diagram of Montelukast-Exposed and -Unexposed Asthma Groups eFigure 2. Flow Diagram of Montelukast-Exposed and -Unexposed Allergic Rhinitis Groups eTable 2. Baseline Characteristics of Patients in Asthma Groups Before and After Matching by Exposure to Montelukast eTable 3. Baseline Characteristics of Patients in Allergic Rhinitis Groups Before and After Matching by Exposure to Montelukast eTable 4. 1-Year Incidence of Sleep Outcomes Before Matching eTable 5. 1-Year Incidence of Mental Health Outcomes Before Matching [file jamanetwopen-e2213643-s001.pdf]

Supplemental Online Content

Paljarvi T, Forton J, Luciano S, Herttua K, Fazel S. Analysis of neuropsychiatric diagnoses after montelukast initiation. *JAMA Netw Open*. 2022;5(5):e2213643.
doi:10.1001/jamanetworkopen.2022.13643

eMethods.

eReferences.

eTable 1. Medical Codes Used in Defining Cohorts, Propensity Score Matching, and Defining Outcomes

eFigure 1. Flow Diagram of Montelukast-Exposed and -Unexposed Asthma Groups

eFigure 2. Flow Diagram of Montelukast-Exposed and -Unexposed Allergic Rhinitis Groups

eTable 2. Baseline Characteristics of Patients in Asthma Groups Before and After Matching by Exposure to Montelukast

eTable 3. Baseline Characteristics of Patients in Allergic Rhinitis Groups Before and After Matching by Exposure to Montelukast

eTable 4. 1-Year Incidence of Sleep Outcomes Before Matching

eTable 5. 1-Year Incidence of Mental Health Outcomes Before Matching

This supplemental material has been provided by the authors to give readers additional information about their work.

© 2022 Paljarvi T et al. *JAMA Network Open.*

eMethods

Data

The HCOs included in the TriNetX Network are typically large academic medical centres that provide a range of healthcare services, including emergency, outpatient, and inpatient care. A single HCO typically has more than one facility, and EHR data from all these facilities are available on the TriNetX platform. Patients are included regardless of insurance status. The quality of TriNetX data is ensured by and evaluated against pre-specified quality standards.[1] The TriNetX data has been used for epidemiological research on various neuropsychiatric outcomes.[2-6]

TriNetX is compliant with the Health Insurance Portability and Accountability Act (HIPAA), the US federal law which protects the privacy and security of healthcare data, and any additional data privacy regulations applicable to the contributing HCO. TriNetX is certified to the ISO 27001:2013 standard and maintains an Information Security Management System (ISMS) to ensure the protection of the healthcare data it has access to and to meet the requirements of the HIPAA Security Rule. Any data displayed on the TriNetX Platform in aggregate form only contains de-identified data as per the de-identification standard defined in Section §164.514(a) of the HIPAA Privacy Rule. The process by which the data is de-identified is attested to through a formal determination by a qualified expert as defined in Section §164.514(b)(1) of the HIPAA Privacy Rule.

Data for this study were accessed via the TriNetX platform and analysed in June 2021 by using the TriNetX built-in query builder. All data processing was conducted using the TriNetX built-in proprietary algorithms. All diagnoses were identified using the International Classification of Diseases, tenth revision, clinical modification (ICD10-CM) codes, while dispensed prescription medicines were identified using the RxNorm codes (eTable 1).

Design

To partially control for potential unmeasured confounding by cohort and period effects in montelukast prescribing and associated factors, we divided the five-year study period, from 1 January 2015 to 31 December 2019, into five separate consecutive one-year blocks based on calendar time. Eligible patients were identified independently within each calendar year, and cohorts were defined and propensity score-matched separately for each calendar year block.

Cohort definitions

In our data, 34% of the patients with dispensed montelukast were prescribed Singulair and 66% generic form montelukast. Patients with missing information were excluded from the analyses. To control for the confounding effect of recent exposure to LTMAs, we excluded patients who had dispensed prescriptions for montelukast, zafirlukast, or zileuton six months before the index prescription. We also excluded patients who had dispensed prescriptions for zafirlukast or zileuton during the follow-up; and additionally, from the control cohorts, those who had dispensed prescriptions for montelukast.

Outcome measurement

Primary outcome measures were 12-month incident neuropsychiatric diagnoses identified by the ICD10-CM codes, including psychotic disorders (F20 – F29); mood disorders (F30 – F39); anxiety, dissociative, stress-related, somatoform and other nonpsychotic mental disorders (F40 – F48); adult personality and behaviour disorders (F60 – F69); sleep disorders (G47, F51); and non-fatal self-harm, which also included events of undetermined intent (T14.91, X71 – X83, Y21 – Y33). Within these broader diagnostic groups we also looked at more specific incident diagnoses, including manic episode or bipolar disorder (F30, F31); major depression, single episode (F32); phobic anxiety (F40); generalized anxiety (F41.1); other anxiety disorders (F41.0, F41.3, F41.8, F41.9); OCD (F42, F60.5; R46.81); insomnia and sleep deprivation (G47.0, F51.0, Z72.820); hypersomnia (G47.1, F51.1); circadian rhythm disorders (G47.2); parasomnias, including sleepwalking, sleep terrors and nightmare disorder (G47.5, F51.3, F51.4, F51.5); sleep related movement disorders and restless legs syndrome (G47.6, G25.81); and other or unspecified sleep disorders (G47.8, G47.9, F51.8, F51.9). By using group-level outcomes and the global outcome, we control for the potential effect of multiple testing, i.e., by testing that the observed associations retain statistical significance at group-level.

For each given primary and secondary outcome, we included patients who did not have a recorded history of the given outcome in their EHR, ie, we modelled incident outcomes. We used a 14-day washout period after the index prescription for measuring outcomes to reduce bias from conditions already present at the time of the index prescription. The EHR data included information on whether the patient had died while in hospital but did not include information on the cause of death. Those who died as inpatients were excluded from the analyses. Due to the lack of cause of death information we were unable to establish whether these deaths could be attributable to montelukast treatment.

Propensity score-matching

We used standardized (mean) differences as a balance metric between the exposed and unexposed cohorts before and after propensity score matching.[7] Montelukast-exposed and -unexposed cohorts of asthma and allergic rhinitis patients were matched for the following covariates: age at index prescription; sex; race; type 2 diabetes mellitus; overweight and obesity; mental and behavioural disorders due to psychoactive substance use; any psychotic disorder; any mood disorder; manic episode; bipolar disorder; major depression, single episode; any anxiety, dissociative, stress-related, somatoform and other nonpsychotic mental disorder; phobic anxiety; generalized anxiety; other anxiety; obsessive-compulsive disorder and

© 2022 Paljarvi T et al. *JAMA Network Open.*

behaviour (OCD); disorders of adult personality and behaviour; any sleep disorder; ischemic heart disease; vasomotor rhinitis; allergic rhinitis due to pollen; other seasonal allergic rhinitis; other allergic rhinitis; unspecified allergic rhinitis; chronic rhinitis; chronic sinusitis; reflux disease; dermatitis and eczema; cough; snoring; self-harm, including undetermined intent; history of dispensed prescriptions for: antihistamines (nasal, oral); sedating antihistamines (promethazine, diphenhydramine, hydroxyzine); non-sedating antihistamines (cetirizine, fexofenadine, loratadine); opioid analgesics; sedatives and hypnotics; antidepressants; antipsychotics; calcium channel blockers; antilipemic agents; ace inhibitors; gastric medications (including medicines used to treat reflux disease); glucocorticoids; antirheumatics; muscle relaxants; decongestants (nasal, systemic); anti-inflammatories (inhalation, nasal, topical); bronchodilators (inhalation, oral, xanthine-derivative, anticholinergic); leukotriene-modifying agents (montelukast, zafirlukast, zileuton); vilanterol; metformin; levothyroxine; antitussives and expectorants; and six most prescribed medications commonly used to treat insomnia (eszopiclone, doxepin, melatonin, temazepam, trazodone, zolpidem). These covariates were included to improve comparability of the montelukast-exposed and control (montelukast-unexposed) cohorts in relation to comorbidities and use of various other prescription medicines that could bias the comparison.

Within the asthma cohort, 76% (n=36245/47772) of montelukast-exposed patients were successfully matched with an unexposed patient. Within the allergic rhinitis cohort, 67% (n=41228/61186) of montelukast-exposed patients were successfully matched with an unexposed patient. The number of excluded patients reflects the fact that in the real-world context, the montelukast exposed and unexposed cohorts, even when these patient groups had the same underlying indication (asthma or allergic rhinitis), had differences in the distribution of potential confounding factors at baseline. This highlights the need to control for these baseline differences as potential confounders.

© 2022 Paljarvi T et al. *JAMA Network Open.*

eReferences

1. Topaloglu U, Palchuk MB. Using a Federated Network of Real-World Data to Optimize Clinical Trials Operations. JCO Clin Cancer Inform. 2018. 2:1-10. doi:10.1200/CCI.17.00067.
2. Paljarvi T, Strang J, Quinn PD, Luciano S, Fazel S. Abuse-deterrent extended-release oxycodone and risk of opioid-related harm. Addiction. 2021. 116(9):2409-2415. doi: 10.1111/add.15392.
3. Harrison PJ, Luciano S. Incidence of Parkinson's disease, dementia, cerebrovascular disease and stroke in bipolar disorder compared to other psychiatric disorders: An electronic health records network study of 66 million people. Bipolar Disord. 2021. 23(5):454-462. doi: 10.1111/bdi.13022.
4. Taquet M, Geddes JR, Husain M, Luciano S, Harrison PJ. 6-month neurological and psychiatric outcomes in 236 379 survivors of COVID-19: a retrospective cohort study using electronic health records. Lancet Psychiatry. 2021. 8(5):416-427. doi: 10.1016/S2215-0366(21)00084-5.
5. Colbourne L, Luciano S, Harrison PJ. Onset and recurrence of psychiatric disorders associated with anti-hypertensive drug classes. Transl Psychiatry. 2021. 11(1):319. doi: 10.1038/s41398-021-01444-1.
6. Taquet M, Luciano S, Geddes JR, Harrison PJ. Bidirectional associations between COVID-19 and psychiatric disorder: retrospective cohort studies of 62 354 COVID-19 cases in the USA. Lancet Psychiatry. 2021. 8(2):130-140. doi: 10.1016/S2215-0366(20)30462-4.
7. Austin PC. An Introduction to Propensity Score Methods for Reducing the Effects of Confounding in Observational Studies. Multivariate Behav Res. 2011. 46(3):399-424. doi:10.1080/00273171.2011.568786.

© 2022 Paljarvi T et al. *JAMA Network Open.*

eTable 1. Medical Codes Used in Defining Cohorts, Propensity Score Matching, and Defining Outcomes

Coding system and codes	Description
ICD-10-CM	*Diagnoses*
C00-D49	Neoplasms
E11	Type 2 diabetes mellitus
E66	Overweight/obesity
F10-F19	Mental and behavioral disorders due to psychoactive substance use
F20-F29	Psychotic disorders
F30-F39	Mood disorders (any)
F30, F31	Manic episode (F30) and bipolar disorder (F31)
F32	Major depression, single episode
F40-F48	Anxiety, dissociative, stress-related, somatoform and other nonpsychotic mental disorders (any)
F40	Phobic anxiety
F41	Generalized (F41.1) and other anxiety
F42, F60.5, R46.81	Obsessive-compulsive disorder and behaviour
F60-F69	Disorders of adult personality and behavior
G47, F51	Sleep disorders (any)
G47.0, F51.0	Insomnia
G47.1, F51.1	Hypersomnia
G47.2	Circadian rhythm disorders
G47.3	Sleep apnea
G47.5, F51.3-F51.4	Parasomnias
G47.6, G25.81	Movement disorders, restless legs syndrome
I20-I25	Ischemic heart disease
J30	Vasomotor and allergic rhinitis
J30.0	Vasomotor rhinitis
J30.1	Allergic rhinitis due to pollen
J30.2	Other seasonal allergic rhinitis
J30.8	Other allergic rhinitis
J30.9	Allergic rhinitis, unspecified
J31	Chronic rhinitis
J32	Chronic sinusitis
J44	Chronic obstructive pulmonary disease (COPD)
J45	Asthma
J45.2	Mild intermittent asthma
J45.3	Mild persistent asthma
J45.4	Moderate persistent asthma
J45.5	Severe persistent asthma
J45.9	Other/unspecified asthma
K21	Reflux disease
L20-L30	Dermatitis/eczema
O00-O9A, Z33	Pregnancy
R05	Cough
R06.0	Dyspnea
R06.83	Snoring
T14.91, X71-X83, Y21-Y33	Self-harm (including undetermined intent)
ICD-10-PCS	*Procedures*
10	Pregnancy
RxNorm/RxCUI	*Medications*
3498	Diphenhydramine
3638	Doxepin
4077	Estazolam
4501	Flurazepam
5553	Hydroxyzine
6711	Melatonin
6809	Metformin
8745	Promethazine
10355	Temazepam
10582	Levothyroxine

(continued)

© 2022 Paljarvi T et al. *JAMA Network Open.*

Coding system and codes	Description
10737	Trazodone
10767	Triazolam
20610	Cetirizine
28889	Loratadine
39993	Zolpidem
40575	Zileuton
74667	Zaleplon
87636	Fexofenadine
88249	Montelukast
114970	Zafirlukast
461016	Eszopiclone
596205	Ramelteon
1424884	Vilanterol
1547099	Suvorexant
AH000	Antihistamines
CN101	Opioid analgesics
CN300	Sedatives/hypnotics
CN600	Antidepressants
CN700	Antipsychotics
CV200	Calcium channel blockers
CV350	Antilipemic agents
CV800	Ace inhibitors
DE200	Anti-inflammatories, topical
GA900	Gastric medications, other (including medicines used to treat reflux disease)
HS051	Glucocorticoids
MS100	Antirheumatics
MS200	Muscle relaxants
NT100	Decongestants, nasal
NT200	Anti-inflammatories, nasal
NT400	Antihistamines, nasal
RE101	Anti-inflammatories, inhalation
RE102	Bronchodilators, inhalation
RE103	Bronchodilators, oral
RE104	Bronchodilators, xanthine-derivative
RE105	Bronchodilators, anticholinergic
RE200	Decongestants, systemic
RE300	Antitussives/expectorants

© 2022 Paljarvi T et al. *JAMA Network Open.*

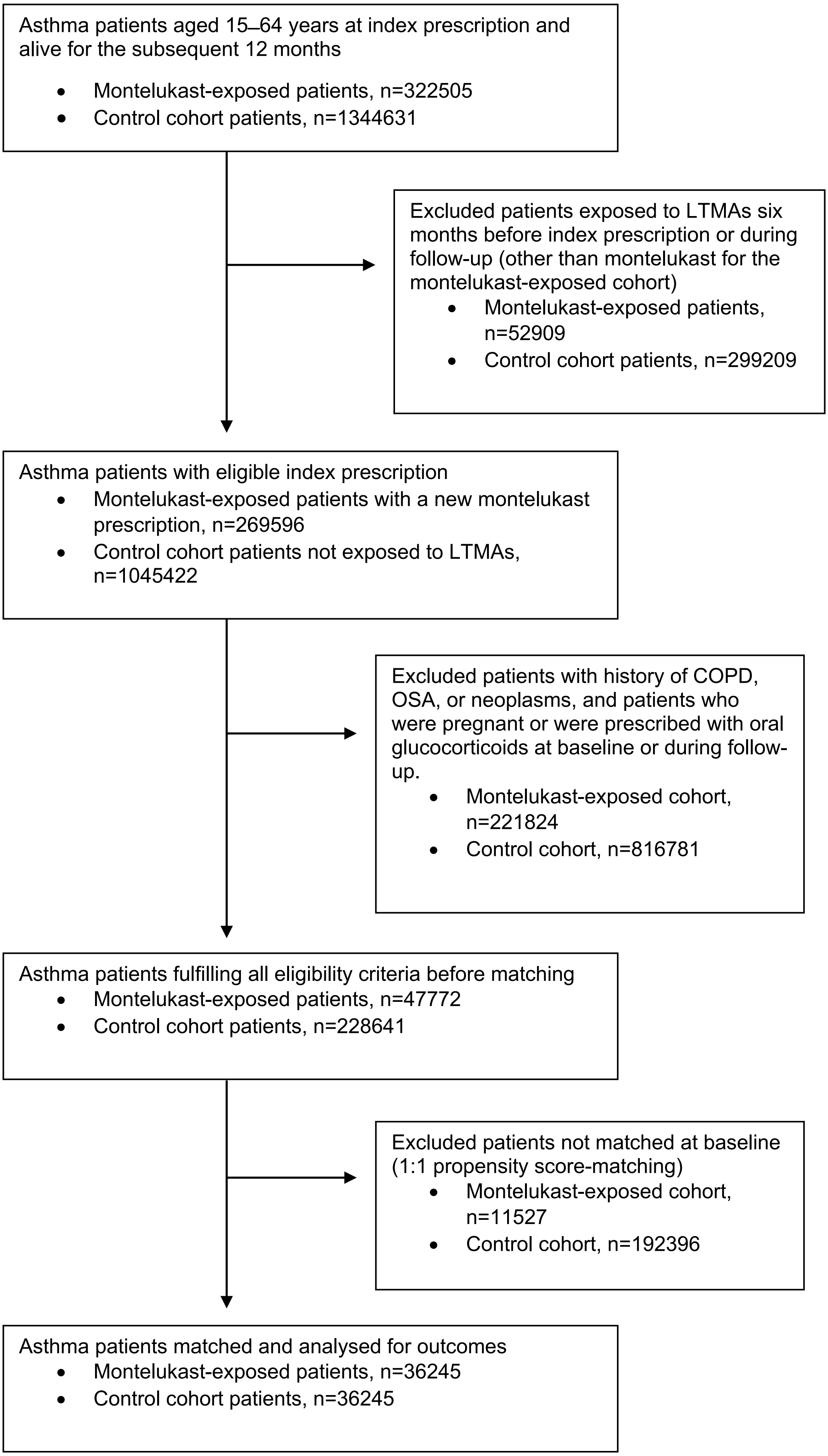

eFigure 1. Flow Diagram of Montelukast-Exposed and -Unexposed Asthma Groups

© 2022 Paljarvi T et al. *JAMA Network Open.*

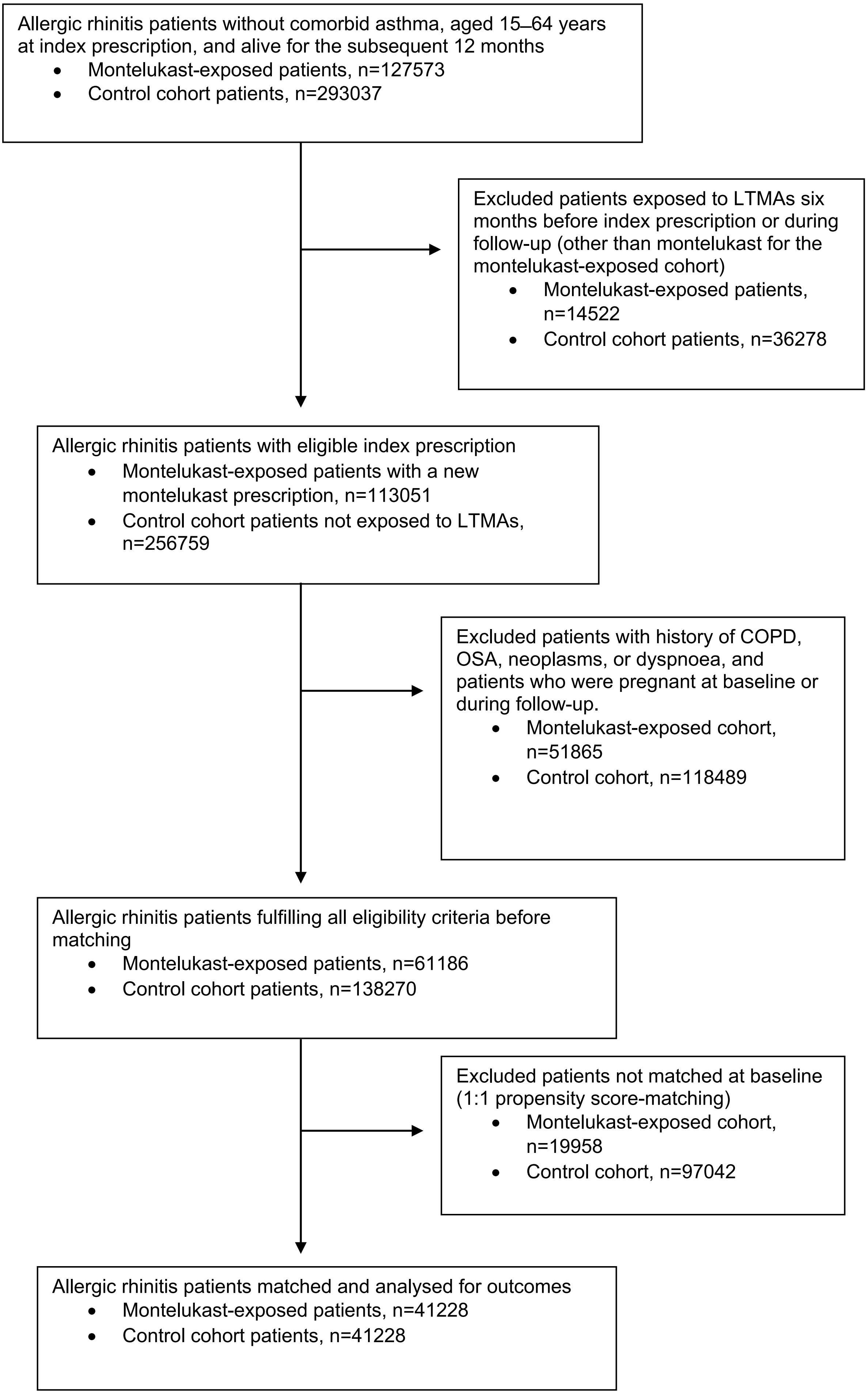

eFigure 2. Flow Diagram of Montelukast-Exposed and -Unexposed Allergic Rhinitis Groups

© 2022 Paljarvi T et al. *JAMA Network Open.*

eTable 2. Baseline Characteristics of Patients in Asthma Groups Before and After Matching by Exposure to Montelukast

	Before matching			After matching		
**Percent**	**Exposed**	**Unexposed**	**Standard difference**	**Exposed**	**Unexposed**	**Standard difference**
Number of patients	47772	228641		36245	36245	
Mean age (SD)	35.9 (15.4)	34.8 (14.9)	0.076	35.1 (15.2)	34.9 (15.2)	0.011
Female	63.0	59.7	0.064	61.6	61.8	0.006
Race, white	70.6	65.6	0.111	68.7	68.4	0.011
Race, black	16.3	21.2	0.129	18.2	18.2	0.009
Montelukast	55.8	7.1	1.225	41.7	41.7	0.001
Zafirlukast	0.1	<0.1	0.025	0.2	0.2	<0.001
Zileuton	0.1	<0.1	0.033	0.1	0.1	<0.001
Asthma						
Mild intermittent	25.7	22.5	0.079	25.6	25.8	0.006
Mild persistent	11.9	7.8	0.120	11.5	11.6	0.010
Moderate persistent	10.3	5.2	0.169	9.4	9.5	0.012
Severe persistent	1.5	0.5	0.081	1.3	1.3	0.006
Other/unspecified asthma	55.8	50.8	0.106	55.8	56.0	0.006
Vasomotor and allergic rhinitis						
Vasomotor rhinitis	21.1	13.3	0.222	21.1	21.4	0.009
Allergic rhinitis due to pollen	10.4	5.1	0.191	8.9	8.9	0.006
Other seasonal allergic rhinitis	11.0	6.6	0.152	10.2	10.2	0.009
Other allergic rhinitis	10.1	4.8	0.194	8.6	8.7	0.009
Unspecified allergic rhinitis	29.6	18.1	0.277	28.0	28.3	0.006
Chronic rhinitis, nasopharyngitis and pharyngitis	5.0	2.9	0.108	4.8	4.9	0.006
Chronic sinusitis	9.8	7.2	0.088	9.5	9.6	0.008
Type 2 diabetes mellitus	6.2	6.5	0.018	6.0	5.8	0.013
Overweight and obesity	14.1	13.8	0.010	14.3	14.1	0.005
Mental and behavioural disorders due to psychoactive substance use	7.6	13.5	0.192	8.9	8.8	0.007
Schizophrenia, schizotypal, delusional, and other non-mood psychotic disorders	1.0	1.9	0.080	1.1	1.1	0.008
Mood (affective) disorders	16.3	18.5	0.060	16.9	16.5	0.013
Manic episode	0.1	0.2	0.015	0.2	0.2	0.008
Bipolar disorder	2.4	3.2	0.049	2.5	2.5	0.011
Major depressive disorder, single episode	12.5	14.3	0.053	13.2	12.8	0.013
Anxiety, dissociative, stress-related, somatoform and other nonpsychotic mental disorders	20.1	21.9	0.047	20.9	20.7	0.009
Other anxiety disorders	17.1	18.4	0.038	17.6	17.6	0.008
Obsessive-compulsive disorder	0.7	0.6	0.010	0.6	0.6	0.010
Sleep disorders not due to a substance or known physiological condition	1.6	1.6	0.011	1.6	1.6	0.012
Disorders of adult personality and behaviour	1.5	2.0	0.037	1.7	1.7	0.009

© 2022 Paljarvi T et al. *JAMA Network Open.*

(continued)

	Before matching			After matching		
**Percent**	**Exposed**	**Unexposed**	**Standard difference**	**Exposed**	**Unexposed**	**Standard difference**
Obsessive-compulsive personality disorder	0.1	<0.1	0.032	0.1	0.1	0.010
Sleep disorders	7.1	7.4	0.013	7.5	7.4	0.004
Ischemic heart diseases	1.7	1.9	0.016	1.7	1.7	0.009
Gastro-esophageal reflux disease	16.5	13.7	0.078	15.8	15.4	0.011
Dermatitis and eczema	12.2	11.7	0.016	13.2	13.4	0.005
Cough	19.3	18.2	0.025	20.1	20.6	0.012
Snoring	5.1	4.4	0.037	5.4	5.5	0.005
Obsessive-compulsive behaviour	0.1	<0.1	0.023	0.1	0.1	0.011
Intentional self-harm	0.2	0.3	0.036	0.2	0.2	0.007
Antihistamines	39.1	32.3	0.139	37.3	37.6	0.006
Diphenhydramine	7.7	9.3	0.060	8.5	8.7	0.009
Hydroxyzine	5.3	6.0	0.029	5.8	5.8	0.003
Promethazine	7.7	8.2	0.022	7.9	7.9	0.005
Cetirizine	16.5	9.9	0.195	15.4	15.4	0.004
Loratadine	10.5	8.3	0.077	10.7	10.8	0.007
Fexofenadine	5.6	3.2	0.115	5.2	5.3	0.005
Opioid analgesics	27.9	31.5	0.084	29.3	29.1	0.006
Sedatives and hypnotics	19.9	21.3	0.039	20.0	19.8	0.010
Temazepam	0.5	0.4	0.013	0.4	0.5	0.007
Zolpidem	3.5	3.6	0.010	3.4	3.5	0.008
Eszopiclone	0.3	0.3	0.012	0.3	0.3	0.005
Antidepressants	24.1	23.8	0.018	23.4	22.7	0.017
Doxepin	0.4	0.4	0.010	0.4	0.4	0.006
Trazodone	4.3	5.1	0.040	4.4	4.3	0.004
Antipsychotics	4.6	6.0	0.063	4.8	4.7	0.006
Calcium channel blockers	5.4	5.3	0.006	5.1	4.9	0.017
Antilipemic agents	10.2	8.7	0.048	8.8	8.3	0.019
Ace inhibitors	7.7	7.7	0.013	7.3	6.9	0.017
Topical anti-inflammatories	53.5	43.4	0.203	50.7	50.9	0.006
Gastric medications (other)	20.1	18.9	0.029	19.4	19.3	0.005
Glucocorticoids	36.1	31.9	0.085	37.0	37.7	0.013
Melatonin	1.7	1.9	0.019	1.8	1.7	0.008
Metformin	4.9	4.5	0.017	4.4	4.2	0.010
Levothyroxine	5.8	4.7	0.048	5.0	4.8	0.011
Antirheumatics	29.3	33.7	0.096	31.3	31.0	0.008
Skeletal muscle relaxants	12.0	12.8	0.029	12.2	12.0	0.010
Nasal decongestants	3.6	3.9	0.015	4.0	4.0	0.007
Nasal anti-inflammatories	54.3	42.1	0.246	51.1	51.3	0.008
Nasal antihistamines	8.3	4.1	0.169	7.2	7.3	0.008
Inhalant anti-inflammatories	52.4	39.8	0.254	49.2	49.4	0.009
Inhalant bronchodilators	66.8	62.5	0.089	63.6	63.3	0.008
Oral bronchodilators	62.3	58.9	0.070	59.8	59.7	0.008
Xanthine-derivative bronchodilators	0.3	0.2	0.016	0.4	0.3	0.009
Anticholinergic bronchodilators	11.5	10.4	0.033	12.1	12.4	0.010
Vilanterol	1.8	0.9	0.064	1.5	1.4	0.009
Systemic decongestants	7.2	6.6	0.026	7.3	7.4	0.005
Antitussives and expectorants	13.5	12.9	0.017	14.0	13.9	0.011
Emergency visit	23.4	29.2	0.131	26.0	25.9	0.004
Inpatient visit	14.9	16.4	0.046	15.4	15.3	0.005

© 2022 Paljarvi T et al. *JAMA Network Open.*

eTable 3. Baseline Characteristics of Patients in Allergic Rhinitis Groups Before and After Matching by Exposure to Montelukast

	Before matching			After matching		
**Percent**	**Exposed**	**Unexposed**	**Standard difference**	**Exposed**	**Unexposed**	**Standard difference**
Number of patients	61186	138270		41228	41228	
Mean age (SD)	40.7 (14.0)	7.8 (14.9)	0.203	40.0 (14.0)	40.3 (14.5)	0.016
Female	66.1	62.6	0.074	65.7	65.7	0.004
Race, white	72.1	58.8	0.287	69.8	70.1	0.008
Race, black	14.3	24.2	0.253	15.4	14.8	0.018
Montelukast	38.5	2.9	0.968	10.1	9.6	0.017
Zafirlukast	0.1	<0.1	0.020	0.1	0.1	<0.001
Zileuton	<0.1	<0.1	0.021	<0.1	<0.1	0.019
Vasomotor and allergic rhinitis						
Vasomotor rhinitis	25.5	21.9	0.109	20.9	20.4	0.012
Allergic rhinitis due to pollen	12.6	7.9	0.142	9.6	9.5	0.011
Other seasonal allergic rhinitis	15.2	14.3	0.034	12.3	12.0	0.010
Other allergic rhinitis	11.5	6.9	0.145	8.4	8.4	0.006
Unspecified allergic rhinitis	38.5	31.7	0.157	32.8	32.5	0.012
Chronic rhinitis, nasopharyngitis and pharyngitis	5.3	3.7	0.081	4.0	4.0	0.005
Chronic sinusitis	17.8	10.6	0.214	14.3	14.6	0.010
Type 2 diabetes mellitus	6.7	7.1	0.015	6.4	6.3	0.008
Overweight and obesity	14.1	15.4	0.045	13.3	13.2	0.006
Mental and behavioural disorders due to psychoactive substance use	6.4	10.4	0.145	6.5	6.3	0.009
Schizophrenia, schizotypal, delusional, and other non-mood psychotic disorders	0.5	1.5	0.103	0.5	0.5	0.004
Mood (affective) disorders	15.4	17.3	0.055	14.5	14.2	0.009
Manic episode	0.1	0.1	0.017	0.1	0.1	<0.001
Bipolar disorder	1.3	2.2	0.065	1.3	1.3	0.008
Major depressive disorder, single episode	12.1	13.5	0.046	11.3	11.1	0.006
Anxiety, dissociative, stress-related, somatoform and other nonpsychotic mental disorders	21.3	20.8	0.006	19.8	19.1	0.015
Other anxiety disorders	18.5	17.2	0.025	17.1	16.7	0.010
Obsessive-compulsive disorder	0.5	0.7	0.019	0.5	0.5	0.008
Sleep disorders not due to a substance or known physiological condition	2.4	1.8	0.031	1.9	1.9	0.004
Disorders of adult personality and behaviour	0.8	1.8	0.088	0.8	0.8	0.015
Obsessive-compulsive personality disorder	0.1	<0.1	0.018	0.1	0.1	0.012
Sleep disorders	8.6	8.3	0.013	7.7	7.6	0.007
Ischemic heart diseases	1.5	1.5	0.008	1.5	1.4	0.009
Gastro-esophageal reflux disease	16.7	16.2	0.012	14.9	14.7	0.007
Dermatitis and eczema	12.2	15.1	0.088	11.3	11.3	0.009
Cough	18.2	15.7	0.063	15.7	15.3	0.009

© 2022 Paljarvi T et al. *JAMA Network Open.*

(Continued)

	Before matching			Before matching		
**Percent**	**Exposed**	**Unexposed**	**Standard difference**	**Exposed**	**Unexposed**	**Standard difference**
Snoring	0.8	0.7	0.008	0.8	0.8	0.005
Obsessive-compulsive behavior	0.1	<0.1	0.018	0.1	<0.1	0.031
Intentional self-harm	0.1	0.1	0.016	0.1	0.1	<0.001
Antihistamines	43.1	55.1	0.245	38.1	35.9	0.046
Diphenhydramine	6.4	10.5	0.152	6.2	6.1	0.005
Hydroxyzine	5.9	7.5	0.065	5.1	5.0	0.010
Promethazine	12.5	10.5	0.061	10.6	10.2	0.014
Cetirizine	14.8	24.5	0.243	13.9	13.4	0.014
Loratadine	9.4	20.9	0.326	9.7	9.9	0.009
Fexofenadine	7.0	8.7	0.060	6.2	6.1	0.006
Opioid analgesics	34.9	35.8	0.022	30.7	29.7	0.021
Sedatives and hypnotics	23.0	23.0	0.008	20.0	19.4	0.014
Temazepam	0.6	0.6	0.008	0.6	0.4	0.016
Zolpidem	4.5	4.1	0.019	3.6	3.6	0.004
Eszopiclone	0.6	0.4	0.018	0.5	0.4	0.007
Antidepressants	26.2	25.4	0.013	22.3	21.7	0.014
Doxepin	0.6	0.5	0.010	0.4	0.4	0.006
Trazodone	4.7	5.5	0.039	4.0	3.9	0.007
Antipsychotics	3.0	5.4	0.123	2.8	2.7	0.012
Calcium channel blockers	6.4	6.8	0.018	5.6	5.4	0.009
Antilipemic agents	14.1	12.7	0.036	11.4	10.9	0.016
Ace inhibitors	10.1	10.0	0.015	8.9	8.6	0.010
Topical anti-inflammatories	53.2	49.7	0.072	45.0	44.5	0.012
Gastric medications (other)	20.9	22.1	0.035	17.8	17.1	0.019
Glucocorticoids	46.4	40.2	0.117	39.8	39.1	0.013
Melatonin	1.0	2.2	0.092	1.0	1.1	0.008
Metformin	5.8	5.8	0.010	5.0	4.9	0.010
Levothyroxine	7.1	5.6	0.060	5.8	5.6	0.009
Antirheumatics	34.8	42.4	0.162	31.9	30.9	0.022
Skeletal muscle relaxants	17.9	17.8	0.008	15.4	14.8	0.020
Nasal decongestants	4.9	6.2	0.062	4.2	4.2	0.011
Nasal anti-inflammatories	49.1	43.5	0.115	40.4	40.0	0.010
Nasal antihistamines	14.0	8.0	0.185	9.1	9.0	0.003
Inhalant anti-inflammatories	43.6	37.5	0.129	35.1	34.7	0.010
Inhalant bronchodilators	20.2	17.2	0.073	15.7	15.3	0.013
Oral bronchodilators	14.5	11.9	0.077	11.2	10.9	0.013
Xanthine-derivative bronchodilators	0.1	0.1	0.009	0.1	0.1	<0.001
Anticholinergic bronchodilators	5.6	5.4	0.010	4.5	4.6	0.003
Vilanterol	0.2	0.1	0.038	0.1	0.1	0.005
Systemic decongestants	11.0	14.0	0.098	9.6	9.4	0.010
Antitussives and expectorants	22.3	20.0	0.049	18.9	18.4	0.011
Emergency visit	20.2	28.9	0.204	20.9	20.2	0.014
Inpatient visit	13.1	16.8	0.105	11.8	11.7	0.004

© 2022 Paljarvi T et al. *JAMA Network Open.*

eTable 4. 1-Year Incidence of Sleep Outcomes Before Matching

	**Patients with asthma**							
	**Exposed (n=47772)**			**Unexposed (n=228641)**				
	**Patients in cohort**	**Patients with outcome**	**IR/1000**	**Patients in cohort**	**Patients with outcome**	**IR/1000**	**OR**	**95%CI**
Any sleep problem	43275	1016	23	206483	4857	23	1.00	0.94, 1.07
Insomnia	44312	768	17	211529	3721	17	0.99	0.91, 1.07
Hypersomnia	47430	113	2	227221	477	2	1.15	0.94, 1.42
Circadian rhythm disorders	47637	55	1	227920	227	1	1.17	0.87, 1.57
Parasomnias	47655	60	1	228013	225	1	1.29	0.97, 1.72
Movement disorders	47328	110	2	226509	498	2	1.07	0.87, 1.31
Other and undefined sleep disorders	46782	237	5	223566	1248	5	0.91	0.80, 1.05
	**Patients with allergic rhinitis**							
	**Exposed (n=61186)**			**Unexposed (n=138270)**				
	**Patients in cohort**	**Patients with outcome**	**IR/1000**	**Patients in cohort**	**Patients with outcome**	**IR/1000**	**OR**	**95%CI**
Any sleep problem	53999	1574	29	123704	3442	28	1.05	0.99, 1.12
Insomnia	55035	1274	23	126579	2718	21	**1.08**	1.01, 1.16
Hypersomnia	60649	138	2	137707	287	2	1.10	0.90, 1.35
Circadian rhythm disorders	60740	52	1	137949	153	1	0.82	0.53, 1.29
Parasomnias	60883	50	1	138173	114	1	1.01	0.73, 1.42
Movement disorders	60291	168	3	137208	314	2	1.19	0.91, 1.56
Other and undefined sleep disorders	60085	338	6	135828	905	7	**0.86**	0.76, 0.97

IR/1000, Incidence rate per 1000 persons; OR, Odds ratio; CI, Confidence interval. Patients aged 15 to 64 years at index prescription in years 2015 – 2019. The same patient can contribute to more than one incident diagnosis from different diagnostic groups during the follow-up.

© 2022 Paljarvi T et al. *JAMA Network Open.*

eTable 5. 1-Year Incidence of Mental Health Outcomes Before Matching

	**Patients with asthma**							
	**Exposed (n=47772)**			**Unexposed (n=228641)**				
	**Patients in cohort**	**Patients with outcome**	**IR/1000**	**Patients in cohort**	**Patients with outcome**	**IR/1000**	**OR**	**95%CI**
Psychotic disorders	47217	94	2	223404	729	3	**0.61**	0.49, 0.76
Mood disorders	38362	1404	36	177601	7424	42	**0.87**	0.82, 0.92
Manic episode/bipolar disorder	46361	197	4	219493	1336	6	**0.70**	0.60, 0.82
Major depression, single episode	40500	1253	31	189399	6646	35	**0.88**	0.83, 0.93
Anxiety and related disorders	36098	2202	61	167993	10499	62	0.98	0.93, 1.02
Phobic anxiety	47327	148	3	226055	694	3	1.02	0.86, 1.22
Generalized anxiety	45089	750	17	214419	3436	16	1.04	0.95, 1.15
Other anxiety	38821	1756	45	182180	8687	48	0.95	0.90, 1.00
Obsessive-compulsive disorder and behaviour	47391	80	2	226946	321	1	1.21	0.95, 1.55
Adult personality disorders	46938	127	2	223395	829	4	**0.74**	0.59, 0.93
Self-harm, non-fatal	47545	56	1	226781	336	1	0.82	0.59, 1.14
	**Patients with allergic rhinitis**							
	**Exposed (n=61186)**			**Unexposed (n=138270)**				
	**Patients in cohort**	**Patients with outcome**	**IR/1000**	**Patients in cohort**	**Patients with outcome**	**IR/1000**	**OR**	**95%CI**
Psychotic disorders	60643	61	1	136222	306	2	**0.46**	0.35, 0.61
Mood disorders	50194	1821	36	110993	4356	39	**0.92**	0.87, 0.98
Manic episode/bipolar disorder	60009	160	3	135000	538	4	**0.68**	0.57, 0.81
Major depression, single episode	52543	1560	30	116965	3802	32	**0.91**	0.86, 0.97
Anxiety and related disorders	45879	3013	66	104986	6481	62	1.07	1.00, 1.15
Phobic anxiety	60477	152	2	137066	423	3	**0.82**	0.68, 0.98
Generalized anxiety	57060	1074	19	130340	2267	17	1.11	0.96, 1.28
Other anxiety	49385	2348	47	113299	5159	45	1.06	0.97, 1.15
Obsessive-compulsive disorder and behaviour	60590	80	1	137346	215	1	0.86	0.66, 1.11
Adult personality disorders	60420	99	2	135745	386	3	**0.59**	0.47, 0.74
Self-harm, non-fatal	60808	50	1	137753	108	1	1.08	0.77, 1.51

IR/1000, Incidence rate per 1000 persons; OR, Odds ratio; CI, Confidence interval. Patients aged 15 to 64 years at index visit in years 2015 – 2019. The same patient can contribute to more than one incident diagnosis from different diagnostic groups during the follow-up.

© 2022 Paljarvi T et al. *JAMA Network Open.*
